# Radiomics Features Differentiate Between Normal and Tumoral High-Fdg Uptake

**DOI:** 10.1038/s41598-018-22319-4

**Published:** 2018-03-02

**Authors:** Chih-Yang Hsu, Mike Doubrovin, Chia-Ho Hua, Omar Mohammed, Barry L. Shulkin, Sue Kaste, Sara Federico, Monica Metzger, Matthew Krasin, Christopher Tinkle, Thomas E. Merchant, John T. Lucas

**Affiliations:** 1 Department of Radiation Oncology, St. Jude Children’s Research Hospital, 262 Danny Thomas Place, Memphis, TN 38105 USA; 2 Department of Diagnostic Imaging, St. Jude Children’s Research Hospital, 262 Danny Thomas Place, Memphis, TN 38105 USA; 3 University of Tennessee Health Sciences College of Medicine, 910 Madison Ave # 1002, Memphis, TN 38103 USA; 4 Department of Oncology, St. Jude Children’s Research Hospital, 262 Danny Thomas Place, Memphis, TN 38105 USA; 5 Department of Radiology, University of Tennessee Health Sciences, Memphis, TN USA

## Abstract

Identification of FDGavid- neoplasms may be obscured by high-uptake normal tissues, thus limiting inferences about the natural history of disease. We introduce a FDG-PET radiomics tissue classifier for differentiating FDGavid- normal tissues from tumor. Thirty-three scans from 15 patients with Hodgkin lymphoma and 68 scans from 23 patients with Ewing sarcoma treated on two prospective clinical trials were retrospectively analyzed. Disease volumes were manually segmented on FDG-PET and CT scans. Brain, heart, kidneys and bladder and tumor volumes were automatically segmented on PET images. Standard-uptake-value (SUV) derived shape and first order radiomics features were computed to build a random forest classifier. Manually segmented volumes were compared to automatically segmented tumor volumes. Classifier accuracy for normal tissues was 90%. Classifier performance was varied across normal tissue types (brain, left kidney and bladder, hear and right kidney were 100%, 96%, 97%, 83% and 87% respectively). Automatically segmented tumor volumes showed high concordance with the manually segmented tumor volumes (R^2^ = 0.97). Inclusion of texture-based radiomics features minimally contributed to classifier performance. Accurate normal tissue segmentation and classification facilitates accurate identification of FDGavid tissues and classification of those tissues as either tumor or normal tissue.

## Introduction

[^18^F] Fluoro-deoxy-glucose positron emission tomography (FDG-PET) is used as a diagnostic tool for cancer staging, prognostication and evaluation of treatment response^1^. While PET has been shown to be sensitive for the detection of neoplastic tissue across multiple tumor types^2–5^, high FDG accumulation and excretion (i.e. brain, heart, kidneys and bladder) in normal tissues routinely precludes standard uptake value (SUV)-based thresholding approaches for identifying tumors^6–9^. In many cases, review of both CT and MR are required for differentiating FDG-avid normal tissues from tumor^10^. Software-assisted methods of differentiating high glucose uptake normal tissues from tumors are needed for facilitating high-throughput analysis of PET studies from clinical trials.

Radiomic feature analysis is a valuable means of evaluating information from FDGavid- tissues^11–15^. The workflow of radiomics analysis includes image segmentation, feature extraction and informatics analysis. Challenges in PET segmentation include image resolution, variability in shape and location of pathologies and image noise^16^. Current techniques of segmenting FDG-PET data are categorized as thresholdbased, stochastic and -learningbased, -regionbased, -boundarybased-, or as jointsegmentation- based. Comparison of PET segmentation techniques is challenging given the lack of standardization. Despite the challenges with auto-segmentation of FDG-avid tissues, quantitative radiomics features have been successfully correlated with disease prognosis and classification^17–20^.

In this study, we retrospectively evaluate the ability of radiomics features derived from SUV and shape data to differentiate FDG-avid normal tissues from tumor tissue using a cohort of Hodgkin lymphoma and Ewing sarcoma patients treated consecutively on two prospective clinical trials. We assess the classification accuracy and identify discriminating radiomics features for differentiating a normal tissue from a tumor. Finally, we investigate additional avenues for improvement to the presented method which have the potential to increase the throughput, accuracy and precision of analysis of FDG-PET studies from clinical trials.

## Methods

### Patient Cohort

Thirty-eight consecutively treated patients were identified on two prospective trials from 2005–2014 and were included in the analysis. Table 1 summarizes the patient information analyzed in this study. Thirty-three [^18^F] FDG-PET/CT scans (FDG-PET) from 15 patients (8 female; median age, 15 y) with Hodgkin disease (HOD) were analyzed, averaging 2.2 scans per patient (Table 1). Sixty-eight FDG-PET scans from 23 patients (10 female; median age 12 y) with Ewing sarcoma (EWS) were collected, averaging 2.9 scans per patient (Table 1).

**Table 1:** Patient information.

	HOD		EWS		Total
	N	%/Median (Range)	N	%/Median (Range)	N
Patients	15	39.5^	23	60.5^	38
Scans	33	32.7^	68	67.3^	101
Male	7	35.0*	13	65.0*	20
Female	8	44.5*	10	55.5*	18
Age (y)	15	15 (6–19)	23	12 (12–23)	38
Height (cm)	15	159 (123–177)	23	156 (156–187)	38
Weight (kg)	15	54 (26–115)	23	53 (53–90)	38

*Calculated proportions are for each indicated disease group, sd = standard deviation, ^Calculated proportions are relative to the entire study population. Legend: HOD = Hodgkin Lymphoma, EWS = Ewing Sarcoma.

This study was reviewed and approved by the St. Jude Children’s Research Hospital Institutional Review Board. All subjects provided informed consent for study participation. All methods were performed in accordance with the relevant guidelines and regulations.

### PET Acquisition Technique

Initially, [^18^F] FDG-PET scan was acquired at the time of diagnosis. The follow-up [^18^F] FDG-PET scans were obtained at different time points during treatment (Supplementary Table 1). Briefly, after fasting at least 4 hours, the patients were administered an intravenous injection of 5.55 MBq/kg (minimum 74 MBq, maximum 444 MBq) [^18^F] FDG. The patients were instructed to urinate before PET/CT scanning to reduce the volume of excreted [^18^F] FDG in the urinary bladder. A non-diagnostic CT scan was performed for attenuation correction and anatomic localization of the lesion before PET/CT image acquisition, which occurred 1 hour after [^18^F] FDG injection. The FDG-PET images were obtained with a GE Discovery LS PET/CT scanner (GE Healthcare, Chicago, IL) set to two-dimensional mode at 3 minutes per bed position for the body and 2 minutes per bed position for the extremities at different resolutions listed in Supplementary Table 2.

### Image Processing

Image intensities (C) are converted to standard uptake values (SUV) based on the patient’s body weight (W), injected dose (D), elapsed time (t) and radionuclide half-life (T_1/2_) in equation (1) with x, y, z as image Cartesian coordinates. Due to the use of different scanners, the original image resolution was anisotropic and inconsistent in the collected scans, as shown in Supplementary Table 2. Cubic image resampling was applied to reach an isotropic resolution with 5 × 5 × 5 mm^3^. After Gaussian filtering for background removal and noise reduction using Equation (2), SUV thresholding with a selected value of 3 was applied in Equation (3) to obtain a binary function I(x, y, z).

1 $$\begin{array}{c}{SUV}(x{,}y{,}z)=\frac{{C}(x{,}y{,}z)}{{2}^{\frac{-{t}}{{{T}}_{1{/}2}}}}\times \frac{{W}}{{D}}\\ \tilde{SUV}(x,y,z)=SUV(x,y,z)\ast G(x,y,z)\end{array},$$

where

2 $$G(x,y,z)=\frac{1}{\sqrt[3]{2\pi }{\sigma }^{3}}\cdot {e}^{-(\frac{{x}^{2}+{y}^{2}+{z}^{2}}{2{\sigma }^{3}})}$$

3 $$I(x,y,z)=\{\begin{array}{cc}1 & \tilde{SUV}(x,y,z)\ge 3\\ 0 & else\end{array}$$

### Tissue Segmentation

After SUV thresholding, FDG-avid regions (brain, heart, kidneys, bladder and tumor) were automatically segmented by using Watershed segmentation^21^ followed by morphological closing^22^. For the HOD cohort, 170 regions were identified from 33 scans, averaging 5.12 regions per scan. For the EWS cohort, 10 of 68 scans were manually segmented because of abnormal uptake in the bones, or super-scans. Altogether, 332 regions were identified from 68 scans, averaging 4.88 regions per scan. Before feature computation, all segmented volumes were reviewed to ascertain accuracy of segmentation. Segmentation performance was evaluated by comparing the segmented volumes with the ground truth established by an assisted algorithm in the treatment planning system. Segmentation performance metrics are reported according to sensitivity, specificity, precision, accuracy, dice similarity coefficient and Jaccard index.

### Radiomics Feature Computation

To build the classifier, radiomics features listed in Supplementary Table 3 were computed for each segmented region, including SUVbased, shapebased and texturebased features. Definitions of each radiomics feature can be found in the paper by Aerts *et al*.^23^. In short, SUV-based features encode information of the SUV distribution in each segmented region. Shape-based features describe 3D size and shape of the region. Six additional shape features describing spatial location and orientation of each segmented region were implemented, including centroid X, centroid Y, centroid X, major axis X, major axis Y and major axis Z. The centroid locations were computed as the center of mass for each segmented region. The major axis was computed as the maximum radius vector.

### Statistical Analysis

Descriptive statistics are reported for all continuous and count data. Continuous data are summarized using the median and range and tested across groups using the Wilcoxon rank sum test. Count data are summarized using frequencies and are tested across groups using either the Fisher’s exact or chi-square test. Feature significance are summarized using student t-tests. Classification accuracy was described using sensitivity and specificity and analyzed with ANOVA. The significance level for statistical tests was p < 0.05 and α = 0.05. Microsoft Excel 2013 (Redmond, WA) was used for all data management. SAS (V 9.3, Cary, NC) or RStudio (V1.0.136, Boston, MA) ggally package were used for analyses. Dimensionality reduction using -tSNE^24^ was used to illustrate the relationship between segmented FDG-avid tissues.

## Results

### Tissue Segmentation

**Figure 1:**
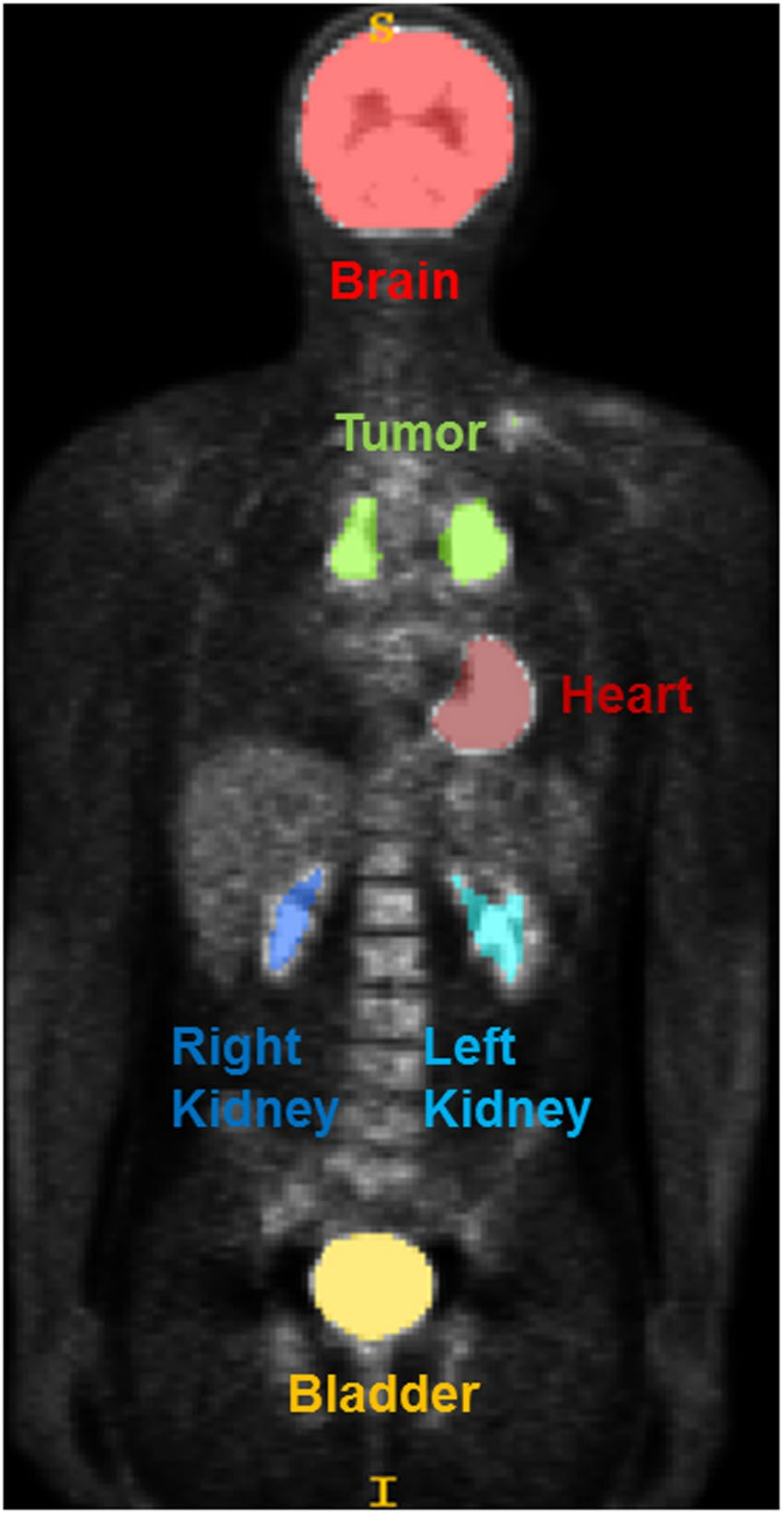
Coronal view of a FDG-PET scan from the time of diagnosis from a 15 year old male with clinical stage IIA nodular sclerosing Hodgkin disease prior to the initiation of chemotherapy with the Stanford V regimen. After image processing and SUV thresholding, FDG-PET avid tissues, such as brain (red), tumor (green), heart (dark red), right kidney (dark blue), left kidney (light blue) and bladder (yellow), are segmented for radiomics feature computation to construct random forest classifiers.

Table 2 lists the total number of regions segmented and their respective ground truth labeling. Among the FDG-avid normal tissues, brain and bladder were present in all HOD and EWS scans. A representative coronal image of a segmented FDG-PET is shown in Fig. 1. Myocardial activity was detected and had uptake >3 in 48 scans (48%). The kidneys were not always apparent as a paired structure: Similarly, left renal activity was visible in 91 scans (91%) and the right kidney, in 86 scans (86%). Tumor was visible in 75 scans (75%). Thyroid and/or salivary glands were only visible in 22 scans (22%), so they were excluded from the tissue classifier. An additional constraint of sole occurrence per tissue in a scan was enforced in classification. Automatic segmentation performance showed a median and 95% confidence interval (CI) of 0.72 (95% CI 0.58–0.82), 0.99 (95% CI 0.98–1.00), 0.78 (95% CI 0.71–0.85), 0.99 (95% CI 0.98–1.00), 0.7 (95% CI 0.63–0.77) and 0.52 (95% CI 0.45–0.59) for sensitivity, specificity, precision, accuracy, dice similarity coefficient and Jaccard index respectively.

**Table 2:** Classifier Performance Results.

Training Set	Test Set Results (Correctly Classified/Manually Segmented)						
	Total	Brain	Heart	Left Kidney	Right Kidney	Bladder	Tumor
HOD NT	284/285 (99.64%)	68/68 (100%)	30/30 (100%)	63/63 (100%)	56/56 (100%)	67/68 (98.5%)	—
HOD NT + T	298/332 (89.8%)	68/68 (100%)	23/30 (76.6%)	48/63 (76.2%)	52/56 (92.9%)	67/68 (98.5%)	40/47 (85.1%)
EWS NT	142/142 (100%)	33/33 (100%)	18/18 (100%)	28/28 (100%)	30/30 (100%)	33/33 (100%)	—
EWS NT + T	152/167 (91.0%)	33/33 (100%)	15/18 (83.3%)	27/28 (96.4%)	26/30 (86.6%)	30/33 (96.9%)	21/25 (84.0%)

HOD = Hodgkin Lymphoma, EWS = Ewing Sarcoma, NT = Normal Tissue, T = Tumor.

### Tissue Classification

**Figure 2:**
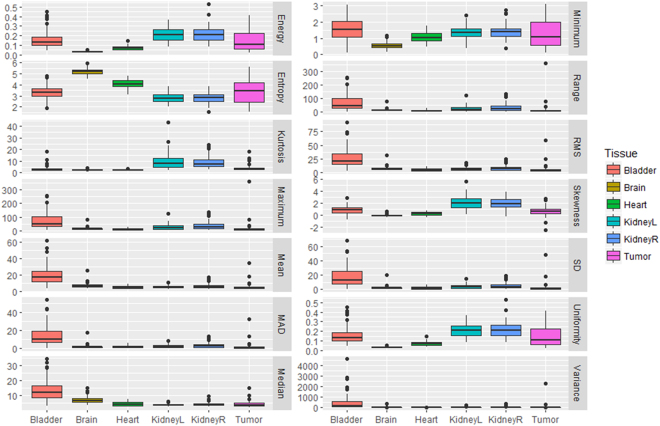
Distribution of 14 SUV-based radiomics features in all segmented volumes. Legend: MAD: Maximum Absolute Deviation, RMS: Root Mean Square, SD: Standard Deviation, KidneyR: Kidney Right, KidneyL: Kidney Left.

**Figure 3:**
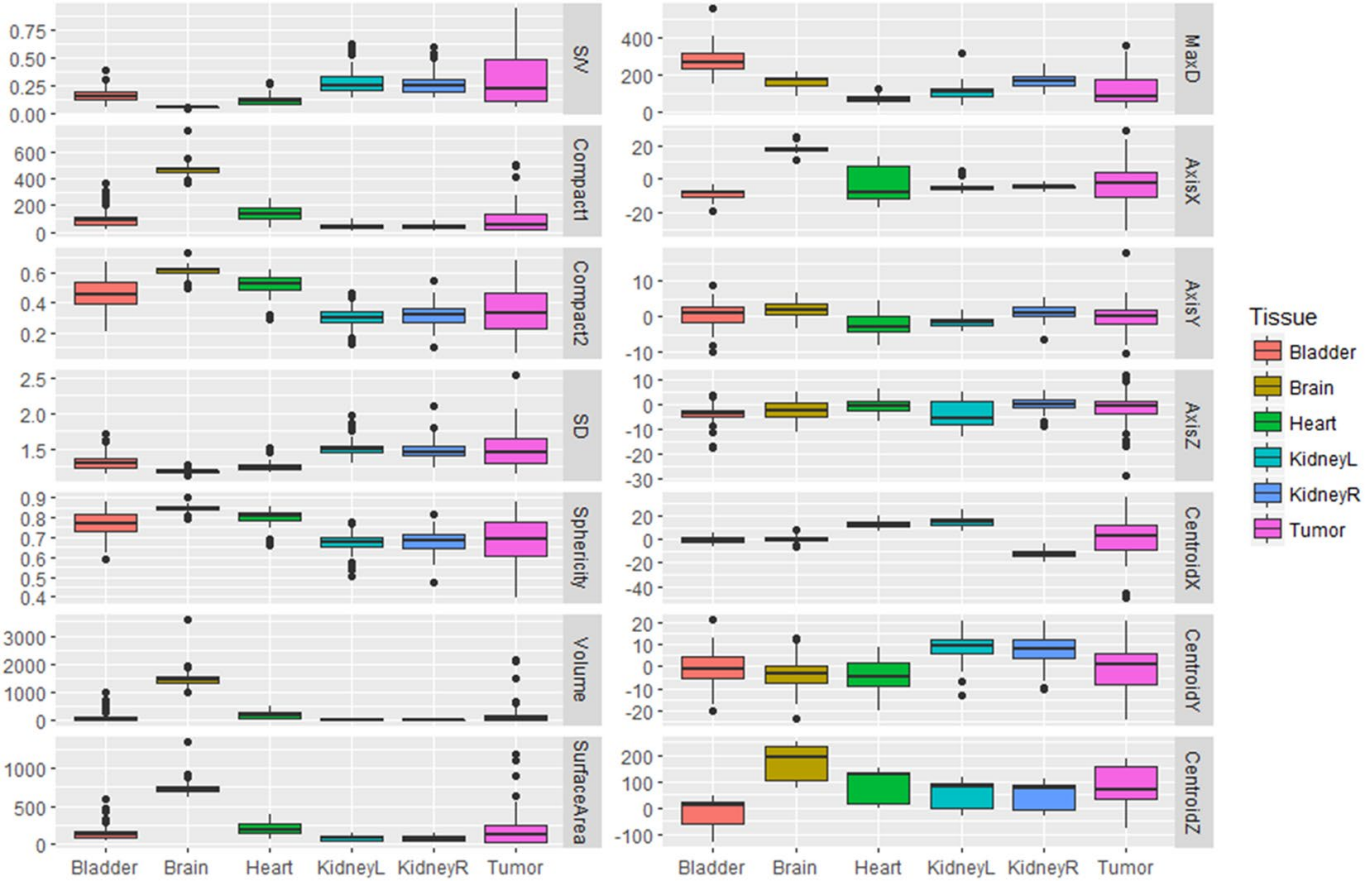
Distribution of 14 shape-based radiomics features in all segmented volumes. Legend: S/V: Surface Volume Ratio, SD: Spherical Disproportion, MaxD: Maximum diameter, KidneyL = Kidney Left, KidneyR: Kidney Right.

**Figure 4:**
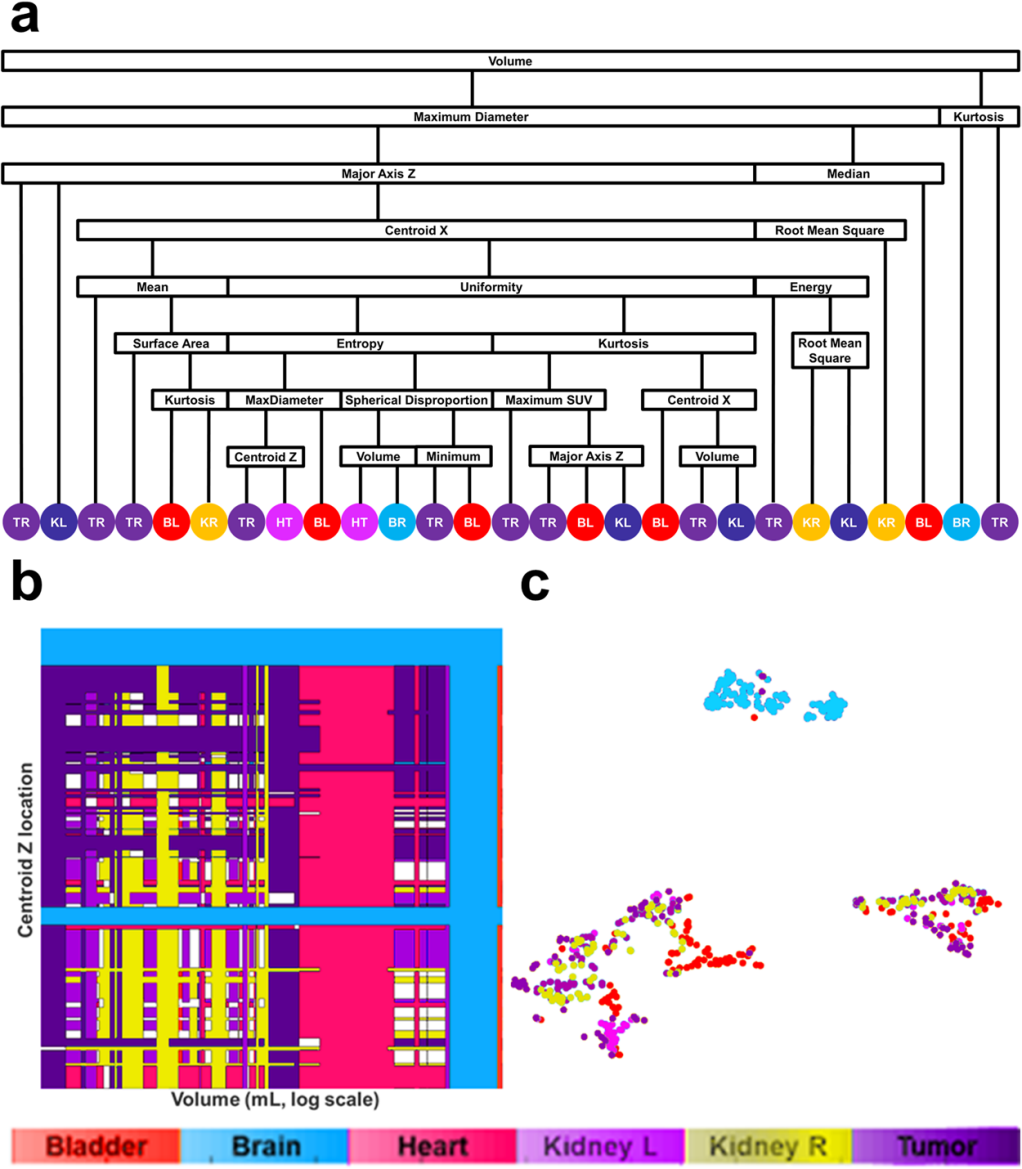
Visualization of random forest classifier. (**a**) Binary decision tree for classifying FDG-avid tissues and tumor. Fifty binary decision trees were trained with the random forest classifier. After traversing the decision tree, tissue classification is decided and annotated. (TR: Tumor, KL: kidney left, BL: bladder, KR: kidney right, HT: heart, BR: brain). (**b**) Display of threshold values for volume and maximum SUV in all 50 trees. The color coding (red: bladder, green: brain, blue: heart, purple: kidney left, gold: kidney right, teal: tumor, white: undecided) shows the tissue classification made on two features: volume and centroid Z. (**c**) t-SNE plot illustrating the classification results for all segmented volumes and features using dimension reduction.

Using the HOD cases as the training data set, a random forest classifier was built with 28 equally weighted radiomics features, as listed in Figs 2 and 3, with uniform prior probabilities. The robustness of the radiomics features was first evaluated by classifying only the normal tissues. The first row in Table 2 summarizes the results for normal tissue classification. There were 285 normal tissue regions from the EWS test set classified and one bladder was mislabeled. Next, the classifier was retrained to include 25 tumor volumes from the HOD scans and re-tested with all 332-normal tissue and tumor volumes from the EWS scans. The classifier correctly identified 298 volumes in the EWS cohort, reaching 89.8% accuracy (Table 2). More specifically, the classifier identified 100%, 98%, 93%, 76%, 76% and 85% of brain, bladder, right kidney, heart, left kidney and tumor tissues (primary site and metastases), respectively. Visualization of the random forest classifier is depicted in Fig. 4. Fifty binary decision trees were trained. Figure 4a shows one binary decision tree and its classification features and results. The final classification result was determined by combinatorial decision among all 50 trees. Figure 4b shows different thresholds computed by all 50 trees in two features: volume and centroid Z location. To better visualize the classification results, we employed t-SNE plots in 4c. It is shown that after dimension reduction, segmented brains are distinctively separate from other FDGavid tissues.

### Classification with EWS as Training Set

To evaluate the influence of sample size, we used the EWS cohort as the training set and the HOD cohort as the test set and built another random forest classifier with the same feature settings. The robustness of the features was again evaluated with 142 normal tissue regions and reached a 100% classification rate (Table 2). We then tested the classifier with 167 segmented normal tissue and tumor regions from the HOD cohort: Increased sample size resulted in improved labeling accuracy for all tissues except for right kidney and bladder (Table 2). In short, the classifier identified 100% of brain, 83% of heart, 96% of left kidney, 87% of right kidney, 97% of bladder and 84% of tumor tissue.

### Validation of Tumor Segmentation

After classification, the automatically segmented tumor volumes were compared with manually segmented volumes drawn by a board-certified radiation oncologist using the CT and the attenuation corrected FDG-PET in MIM Software Inc. (Version 6.7, Beachwood, OH). Supplementary Fig. 1 shows the correlation between manually delineated and segmented tumor volumes. The determination coefficient demonstrates the high consistency of the automatic segmentation method relative to the manual segmented volumes (R^2^ = 0.97). The first-order coefficient 1.06 indicates that segmentation slightly overestimates tumor volume. The constant offset of −3.5 indicates that automatic segmentation may not identify small lesions (<4 ml).

### Misclassified Regions

**Figure 5:**
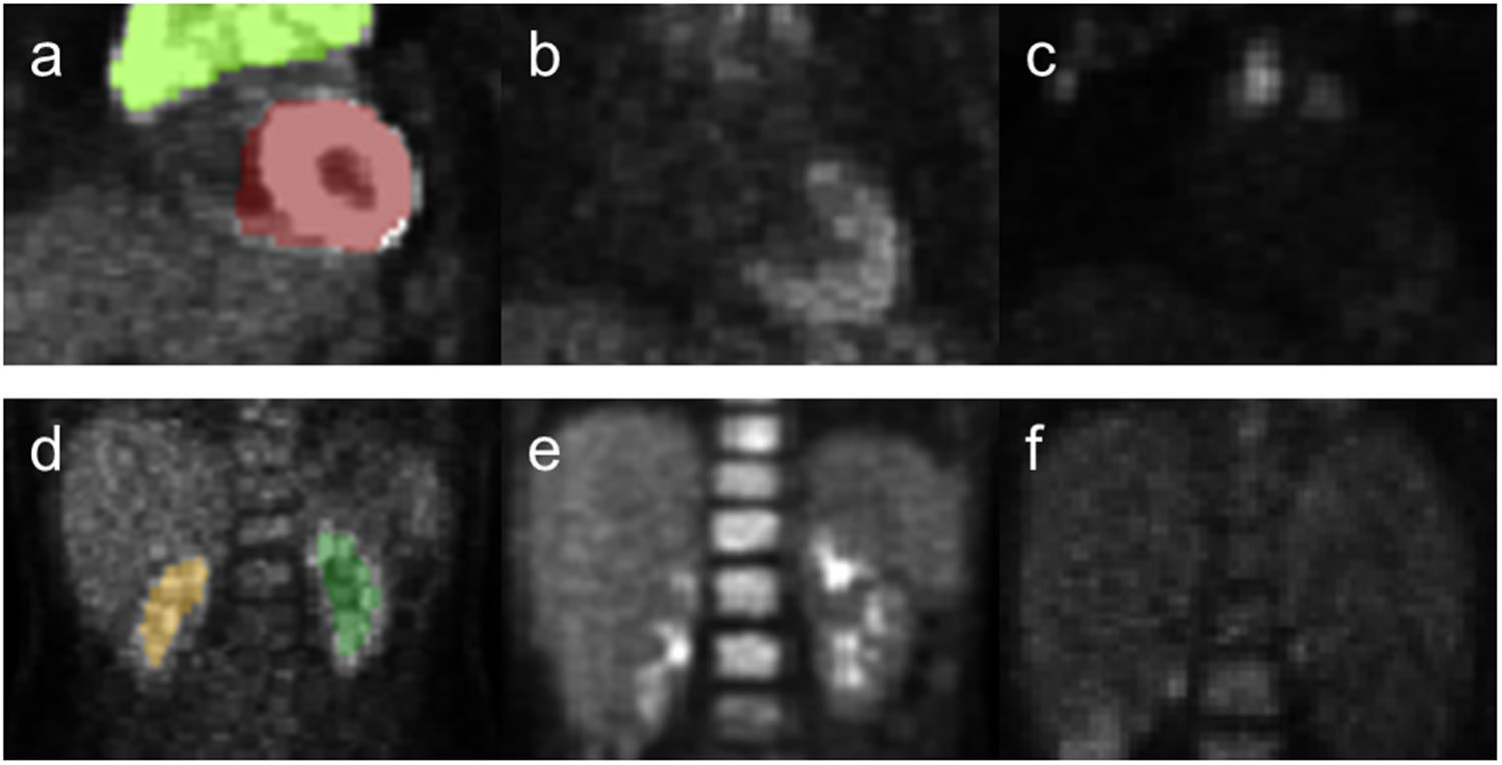
Various tissue uptake of heart (**a**–**c**) and kidney (**d**–**f**) in FDG-PET. (**a**–**c**) In three different scans, the heart exhibits left ventricular and left atrial uptake (**a**), left ventricular uptake (**b**) and no detectable uptake (**c**). Segmentation results overlaid on the scans demonstrates the effect of partial tissue uptake. (**d**–**f**) Similar behavior can also be observed in the kidneys, as exhibited by activity in the cortex and collecting system (**d**), activity in the collecting system (**e**) and absence of activity in (**f**).

Figures 2 and 3 illustrate the respective distribution of all SUVbased and shapebased features. Recognition of the brain when training with either dataset reached 100% because of the large volume of this organ relative to other tissues, as shown in Fig. 3 (brain, bladder, heart, left kidney, right kidney and tumor average volumes ± standard deviation were 1468.37 ± 266.15 mL, 118.94 ± 153.07, 197.59 ± 125.63, 30.70 ± 22.61, 31.20 ± 22.85 and 193.90 ± 394.46 respectively; p < 0.001). Bladder and tumor volumes were at the lower end of the range of brain volumes but could be differentiated from brain by using other features, such as compactness 1. Heart misclassification posed a unique challenge, regardless of the training dataset. This may be due to insufficient samples and inconsistent FDG uptake in the heart, which often lead to varied segmentation patterns, as demonstrated in Fig. 5. Across all scans, 48 exhibited high uptake, with 36 displaying the entire heart and 12 displaying only the apex. The recognition rate improved from 76% to 83% after training with EWS cohort. Three hearts remained misclassified due to the existence of tumors with similar shape. As shown in Fig. 2, only entropy could differentiate heart from other normal tissues and tumor (p < 0.001, α = 0.05). Among shapebased features, compactness 1 and compactness 2 were useful for differentiating heart from other normal tissues (p < 0.001 for each) and from tumors (p < 0.005).

The respective recognition rates of left and right kidneys were 96% and 86%, with one and four misclassifications, respectively. As shown in Fig. 2, kurtosis and skewness (p < 0.001 for each) were useful SUV-based radiomics features for identifying kidneys. Centroid Y (posterioranterior) also had high distinctive power between kidneys and other FDGavid tissues. For distinguishing between left and right kidneys, only the Centroid X location had high distinctive power (p < 0.001).

High FDG uptake, indicated by maximum SUV, mean SUV, median SUV, root mean square and mean absolute deviation, significantly distinguished bladder from other FDGavid- tissue, as shown in Fig. 3 (p < 0.001). Moreover, centroid location in the superiorinferior- direction (Centroid Z) also distinguishes bladder from other normal tissues along the z axis as bladder is typically the lowest segmented tissue in the scans. Given the inter-individual differences in patient height, only relative location could be obtained and used in the tissue classifier. The variable scan parameters used for PET scans among institutions also complicate the use of the centroid z metric. Among HOD and EWS scans, 8 and 64 scans were full-body and 25 and 4 scans encompassed only head to pelvis. Potential solutions to centroid z inconsistencies include height normalization, standardization of scan length and image alignment.

### Classification Power of Radiomics Features

Table 3 lists the top five features with the most discriminatory influence in the tissue classifier. Shape features, such as volume and compactness, were useful for distinguishing brain from tumor. A similar trend was noted for heart-tumor comparisons. Kidney identification was improved with the inclusion of SUV-based features, such as skewness and kurtosis. Only one shape feature, Centroid Z, added to the discriminatory capability of the classifier for bladder vs. other tissue.

**Table 3:** Most-discriminatory Radiomics Features for Each Tissue Type.

Tissue	Brain	Heart	KidneyL	KidneyR	Bladder
Feature 1	Volume	Sphericity	Skewness	Skewness	Median
Feature 2	Compactness 1	Compactness 2	Centroid Y	Kurtosis	Mean
Feature 3	Surface Area	Spherical Disproportion	Kurtosis	Centroid Y	Root Mean Square
Feature 4	Entropy	Surface Volume Ratio	Centroid X	Centroid X	Mean Absolute Deviation
Feature 5	Compactness 2	Energy	Entropy	Entropy	Centroid Z

### Inclusion of Texture Features

We then evaluated the ability of texture-based features extracted from the SUV measurements (including gray level cooccurrence matrix and gray level run length matrix) to add to the discriminatory power of the tissue classifier.

Supplementary Table 3 lists the 34 texture features computed. Definitions for each texture-based feature can be found in Aerts *et al*.^23^. The overall performance increased by 0.6%, with only two more hearts being correctly identified. To accommodate for the additional features, the number of trees was doubled compared to previous classifiers. The overall performance did not improve with an associated increase in the number and complexity of trees, indicating that the classifier had reached its performance limitation with an outofbag error of 0.06.

## Discussion

Accurate tissue segmentation is essential for radiomics feature computation. Although thresholding methods have limitations, several studies^25–27^ have proposed iterative thresholding for accurate PET segmentation. In this study, SUV thresholding of 3 with watershed segmentation was shown to have both high specificity in FDG-PET imaging and an excellent coefficient of determination relative to manual tissue segmentation. Similar techniques achieve comparable segmentation performance in PET phantom studies^28^.

Tissues with inconsistent uptake and anatomical heterogeneity, such as brain and heart, may be over-segmented. In brain, SUV may vary across grey matter (high SUV) relative to white matter (low SUV). Analogously, in heart, the left ventricle has a higher SUV than the right ventricle and atria. To overcome the possibility of over-segmentation and under-segmentation, we applied image resampling and Gaussian smoothing to provide a more-uniform SUV across the entirety of the segmented organ. This approach has been reported to improve the spatial resolution of radiopharmaceutical uptake estimates and the signal-to-noise ratio of FDG-PET^29^.

Conversely, infrequent abnormal uptake in normal tissues may lead to spurious undersegmentation due to unique circumstances. We observed this in the context of abnormal large intestine uptake leading to undersegmentation of the intestine and bladder. Undersegmentation of these structures may be remedied by applying automatic Otsu thresholding^30^ to adjust the local SUV threshold.

Partial and/or variable patterns of FDG uptake in normal tissues had minimal negative impact on classifier performance. Kidney and heart FDG avidity are examples of this phenomenon and representative images are shown in Fig. 5. Although this issue may lead to incomplete segmentation, our classifier performance was only minimally impacted. To improve kidney segmentation, computed tomography (CT)-based segmentation techniques can be incorporated^31–33^ by transferring the segmented masks to the co-registered CT-based attenuation correction (CTAC) scans completed at the time of PET acquisition. Although CTAC studies frequently lack the resolution of diagnostic CTs for each body region, they likely contain enough information about each corresponding tissue to provide additional information to add to iterative image segmentation methods to limit the impact of partial segmentation due to varied FDG-uptake. Ramos *et al*.^34^ have also suggested using iterative image reconstruction as a method to achieve uniform SUV in FDGavid tissues. While normal organ volumes often under estimated the total organ size and volume, this was not the purpose of our methodology and FDG-avid regions were characterized for the sole purpose of differentiating them from tumor.

Feature stability should also be considered when selecting radiomics features for use as classifiers^31^. Shinohara *et al*.^35^ demonstrated that intensity normalization in MR may enable multimodality comparisons and longitudinal evaluation of MR studies. These methods may also improve the stability of MR- and PET-derived radiomics features. Additional methods, such as height normalization, voxel dimension standardization, PET acquisition standardization and SUV normalization^36^, may stabilize FDGPET radiomics features and improve classifier performance and FDG-avid tissue segmentation. Successful separation of normal tissue and tumor can facilitate volume delineation for radiotherapy planning and tracking tumor response and disease burden^37^.

Tumor segmentation accuracy was excellent using our classifier. Manually segmented tumor volumes showed a high correlation with automatically segmented tumor volumes with an R^2^ of 0.97. Segmentation performance was superior in cases with solid tumors compared to nodular cases (ANOVA, p = 0.01). Incorporation of CT images and local thresholding may potentially improve classifier performance. Limitations in CT z-plane resolution were partially overcome by resampling of the CTs to 0.25 × 0.25 mm in the treatment planning system thus aiding manual segmentation to a reasonably level of accuracy. Additional inaccuracies in manual delineation were secondary to region directed but not tumor directed CT scans. In some cases, tumor would span the head and neck region down into the chest resulting in partial overlap and registration difficulties. Automatically segmented tumor based on FDG-PET was completed on a per scan basis, thus overcoming any potential errors in registration or partial overlap across studies. Future studies comparing different tumor types will need to carefully evaluate the relationship between radiotracer avid tumor and tumor heterogeneity to avoid over or under estimating tumor burden. Additional studies in other tumor systems are required to determine whether other tumor types have divergent enough radiomic feature profiles to facilitate accurate differentiation of normal tissue and tumor.

In this study, texture features in FDGPET appear to have low discriminating powers between normal FDGavid tissues and tumor. Nevertheless, texture analysis in CT and MRI has been demonstrated to facilitate differentiating tumor behavior and response^38–43^. The varied reproducibility of each feature across studies confounds the utility of textural features^44,45^. Future large-scale studies should continue to evaluate the longitudinal stability of PET- and CT-derived radiomics features in normal tissues to reduce the likelihood that excessive radiomic feature number will over-fit predictive prognostic models. Furthermore, the proposed image processing pipeline facilitated segmentation of the entire organ despite the nonuniform signals within kidneys and heart. This work might potentially extend to segment tumor volumes in other diseases or alternative PET radiotracer studies^46^. The classifier has been made available to public at https://goo.gl/LzJyjY with pseudocodes shown in Supplementary Fig. 2.

## Conclusion

This study utilized SUV-based thresholding for FDG-avid tissue identifications followed by radiomics feature extraction and a random forest-based machine learning to develop a classifier that could distinguish FDGavid normal tissues from tumor. We achieved an overall 90% classification rate using predominately shape-based radiomics features for identifying tumor and normal tissues. Normal tissues that could be identified in a reproducible manner based on first-order radiomics features alone derived from SUV data include bladder, heart, kidneys and brain. SUV textural features did not improve classifier performance. Application of such SUV-based iterative thresholding methods with subsequent tissue classification could aid in the analysis and interpretation of automatically segmented FDG-avid tumor regions on longitudinal PET studies from clinical trials by removing the confounding contribution of FDG-avid normal tissues from the analysis.

## Electronic supplementary material

Supplementary Information
