# Supplementary material for: Radiomics Features Differentiate Between Normal and Tumoral High-Fdg Uptake
Source: Sci Rep. 2018 Mar 2;8:3913. doi: 10.1038/s41598-018-22319-4 (PMC5834444; doi:10.1038/s41598-018-22319-4)
Supplement: Supplementary file 1 — Supplementary Information [file 41598_2018_22319_MOESM1_ESM.docx]

**RADIOMICS FEATURES DIFFERENTIATE BETWEEN NORMAL AND TUMORAL HIGH-FDG UPTAKE Supplementary**

Chih-Yang Hsu^1*^, Mike Doubrovin^2^, Chia-Ho Hua^1^, Omar Mohammed^3^, Barry L. Shulkin^2^, Sue Kaste^2,4,5^, Sara Federico^4^, Monica Metzger^4^, Matthew Krasin^1^, Christopher Tinkle^1^, Thomas E. Merchant^1^, John T. Lucas Jr.^1^

^1^Department of Radiation Oncology, St. Jude Children’s Research Hospital, 262 Danny Thomas Place, Memphis, TN 38105

^2^Department of Diagnostic Imaging, St. Jude Children’s Research Hospital, 262 Danny Thomas Place, Memphis, TN 38105

^3^University of Tennessee Health Sciences College of Medicine, 910 Madison Ave # 1002, Memphis, TN 38103

^4^Department of Oncology, St. Jude Children’s Research Hospital, 262 Danny Thomas Place, Memphis, TN 38105

^5^Department of Radiology, University of Tennessee Health Sciences, Memphis, TN

**Keywords:** Positron Emission Tomography, Radiomics, Classifier, Automatic Segmentation

**Corresponding Author**: Chih-Yang Hsu, Department of Radiation Oncology, St. Jude Children’s Research Hospital, 262 Danny Thomas Place, MS 210, Memphis TN 38105, phone: 901-595-1119, fax: 901-595-3130, chih-yang.hsu@stjude.org

**Supplementary Tables**

Supplementary Table 1. PET scans collected at different time points

|  | HOD | EWS |
| --- | --- | --- |
| At Diagnosis | 15 | 23 |
| 60 days | 11 | 16 |
| 120 days | 3 | 10 |
| 180 days | 2 | 8 |
| 240 days | 0 | 4 |
| 300 days | 1 | 2 |
| 360 days | 1 | 2 |
| 720 days | 0 | 3 |

Supplementary Table 2. Image resolution of collected scans

| Image Resolution (mm^3^) | Number of Scans |
| --- | --- |
| 2.60×2.60×3.27 | 4 |
| 3.90×3.90×4.25 | 33 |
| 3.65×3.65×3.27 | 62 |
| 4.07×4.07×5.00 | 1 |
| 5.30×5.30×3.37 | 1 |

Supplementary Table 3. Radiomics Features Utilized for FDG-Avid Tissue Classifier

| Feature Type | | Radiomics Feature List |
| --- | --- | --- |
| SUV-based | | Energy, Entropy, Kurtosis, Maximum, Mean, Mean Absolute Deviation, Median, Minimum, Range, Root Mean Square, Skewness, Standard Deviation, Uniformity, Variance |
| Shape-based | | Surface Volume Ratio, Compactness 1, Compactness 2, Spherical Disproportion, Sphericity, Surface Area, Volume, Centroid X, Centroid Y, Centroid Z, Maximum Diameter, Major Axis X, Major Axis Y, Major Axis Z |
| Texture-based | Gray Level Co-Occurrence Matrix | Auto Correlation, Cluster Prominence, Cluster Shade, Cluster Tendency, Contrast, Correlation, Difference Entropy, Difference Variance, Dissimilarity, Energy, Entropy, Homogeneity 1, Homogeneity 2, Informational Measure of Correlation 1, Informational Measure of Correlation 2, Inverse Difference Normalized, Inverse Difference Moment Normalized, Inverse Variance, Maximum Probability, Sum Average, Sum Entropy, Sum Variance, Variance, Run Length Non-uniformity |
|  | Gray Level Run Length Matrix | Short Run Emphasis, Long Run Emphasis, Gray Level Non-uniformity, Run Percentage, Low Gray Level Run Emphasis, High Gray Level Run Emphasis, Short Run Low Gray Level Emphasis, Short Run High Gray Level Emphasis, Long Run Low Gray Level Emphasis, Long Run High Gray Level Emphasis |

**Supplementary Figure**

| 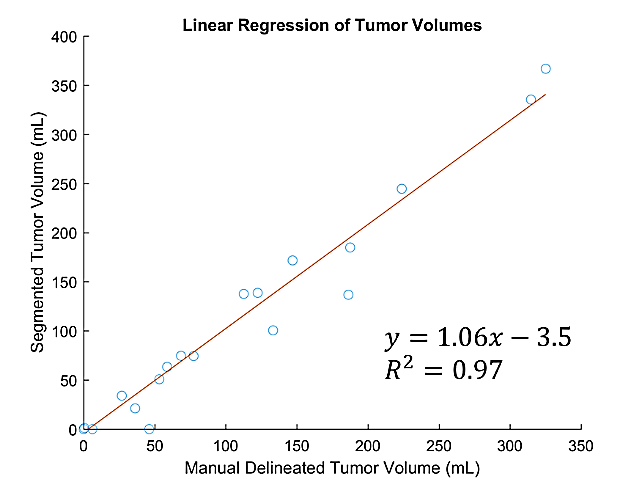  Supplementary Figure 1. Linear regression of manual delineated and segmented tumor volumes. The determination coefficient R^2^=0.97 demonstrates good correlation between manually delineated and segmented volumes. |
| --- |

|  |
| --- |


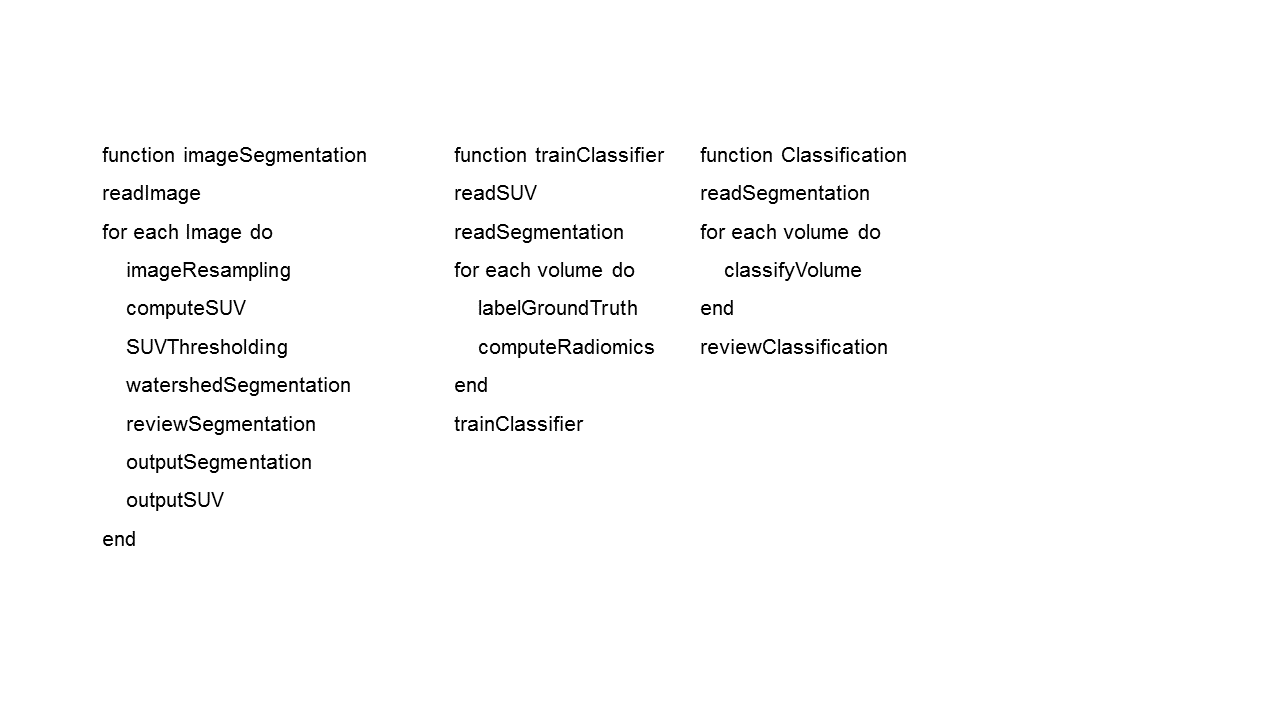


Supplementary Figure 2. Pseudocode for FDG-PET classifier.
